# Processing Aspectual Agreement in an Inflexionless Language: An ERP Study of Mandarin Chinese

**DOI:** 10.3390/brainsci11091236

**Published:** 2021-09-18

**Authors:** Yuxin Hao, Xun Duan, Lu Zhang

**Affiliations:** 1 Institute of Chinese Language and Culture Education, Huaqiao University, Xiamen 361021, China; 2 College of Chinese Language and Cultutre, Huaqiao University, Xiamen 361021, China; 19014041006@stu.hqu.edu.cn (X.D.); 18014041033@stu.hqu.edu.cn (L.Z.)

**Keywords:** lexical aspect, aspect marker, prediction, Chinese, ERP

## Abstract

This is a study of the collocation of Chinese verbs with different lexical aspects and aspect markers. Using event-related potentials (ERPs), we explored the processing of aspect violation sentences. In the experiment, we combined verbs of various lexical aspect types with the progressive aspect marker *zhe*, and the combination of the achievement verbs and the progressive aspect marker *zhe* constituted the sentence’s aspect violation. The participants needed to judge whether a sentence was correct after it was presented. Finally, we observed and analyzed the components of ERPs. The results suggest that when the collocation of aspect markers and lexical aspect is ungrammatical, the N400-like and P600 are elicited on aspect markers, while the late AN is elicited by the word after the aspect marker. P600 and N400-like show that the collocation of Chinese verbs with various lexical aspects and aspect markers involve not only syntactic processing, but also the semantic processing; and the late AN may have been due to the syntax revision and the conclusion at the end of sentences.

## 1. Introduction

Tense and aspect are important means for human language to encode time information [1]. Tense refers to the time when an action takes place, such as the present, past or future [2]. Aspect refers to different ways of viewing the temporal characteristics of a situation [3]. There are two types of aspect: “grammatical aspect” and “lexical aspect” [4]. Grammatical aspect refers to a state or a series of progressive states observed by the speaker in a time viewpoint or a series of time viewpoints [5,6], that is, whether the event is in progress or has been completed. Grammatical aspect is generally divided into perfect aspect (e.g., (1a), expressed by “has + v-ed”) and progressive aspect (e.g., (1b), expressed by “is + v-ing”). The perfect aspect views a situation from the outside, and presents an indivisible whole, i.e., a completed or stopped event; while the progressive aspect views a situation from the inside, i.e., the presented situation is an ongoing process, which is usually continuous and implies temporality, dynamism and willingness [7]. According to the concept of grammatical aspect and Smith’s [7] description of “perfect aspect” and “progressive aspect”, grammatical aspect is categorized as syntax. In Indo-European languages, “grammatical aspect” is usually expressed by verbs’ morphological changes (for English, this is exemplified in (1)):

**Table:** 

(1)	a. So far, John has learned his courses.
	b. Right now, John is learning his courses.

However, Chinese, and other languages which lack morphological changes, use aspect markers to express “grammatical aspect”, such as (2a) in which the aspect marker le indicates the completion of the action: (2a) using *zhe* to indicate that the action is in progress.

**Table:** 

(2)	a. 安娜 到 了 北京。
	Anna dao *le* Beijing
	Anna arrive-*le* Beijing
	Anna has been to Beijing.
	b. 安娜 看 着 比赛。
	Anna kan *zhe* bisai
	Anna watch-*zhe* bisai
	Anna is watching the game.

Lexical aspect refers to situation types denoted in the verb (phrase) that are distinguished on the basis of temporal properties, such as dynamism, durativity and telicity [4]. The lexical aspect is correlated with the time characteristics of the situation described by the verb, and it is the intrinsic semantic feature of the verb or verb phrase [8,9]. According to the semantics of verb and time structure, Vendler [10] distinguished four major lexical aspect categories, or verb types—state, activity, accomplishment and achievement. Andersen et al. [11] made a detailed statement which supplemented Vender’s [10] lexical aspect categories. They believed that state verbs express state and describe a persistent situation. Unless affected by an external situation, they do not change, and maintain continuity, such as have, think, believe or be (tall, healthy). The activity verbs express homogeneous actions that do not affect the results. They describe a dynamic and persistent situation, and the end of the situation is arbitrary, that is, the situation can be terminated at any time, such as sing, write or walk. The accomplishment verbs describe a dynamic and persistent situation, but unlike the activity verbs, this situation has a natural end. After this end, the specific action cannot continue, that is, it has finality, such as writing a letter or building a house. Achievement verbs describe an instant situation at a point in time, which also has a natural ending. However, unlike the accomplishment verb, this action occurs in an instant, and the verb or verb phrase itself has indicated the result, such as arrive, leave or notice somebody. Thus, the lexical aspect of a verb is correlated with its semantic meaning.

Previous researchers have explored the processing of time reference words (time nouns or time adverbs) and grammatical aspect violation in sentences (e.g., [12,13,14,15]). However, there is no research on the collocation processing of the lexical aspect of verbs and aspect markers. The purpose of this study is to explore the processing of lexical aspect of verbs and aspect markers without morphological changes in Chinese, and to compare and contrast the processing between Chinese aspect and Indo-European languages’ aspect. Before outlining the design of this study, we summarize previous ERP research on temporal information processing.

### 1.1. ERP Studies on Aspect

ERPs (event-related potentials) pertain to cognitive function. ERPs describe psychological activities such as recognition, comparison, judgment, memory and decision on the basis of attention. They reflect various aspects of the cognitive process, and are a window into the brain’s cognitive function activities. The ERP components which pertain to language processing are N400, P600 and LAN. N400 is a negativity with a latency of 300–600 ms distributed throughout the central parietal region, the posterior part of the brain and the left and right sides of the brain. N400 is found in semantic disagreement sentences. Many scholars of psychophysiology believe that N400 is involved in semantic processing [16,17,18]. P600 is a positivity, most prevalent in the posterior part of the brain, with peaks appearing at about 500–900 ms. It is also sensitive to the complexity of morphology, syntactic information and syntax [19]. LAN between 300–500 ms is caused by morphosyntactic violation [20] (Table 1).

With the emergence of ERPs and other technical means, more research on the cognitive processing of aspect is being conducted. As shown from example (3), “has + learned” means present perfect, “had + learned” means past perfect, “is + learning” means present progressive, and “was + learning” means past progressive.

**Table:** 

(3)	a. So far, John has learned his courses.
	b. By the time he came, John had learned his courses.
	c. Right now, John is learning his courses.
	d. This morning, John was learning his courses.

As aspect is often associated with temporal context, researchers have used the mismatch between temporal words and aspect to construct the aspect violation paradigm, and have used the violation paradigm to study aspect processing. This is exemplified in (4).

**Table:** 

(4)	a. So far, John has listened the video.
	* Tomorrow, John has listened the video.
	b. By the time he came, John had completed his homework.
	* Tomorrow, John had completed his homework.
	c. Right now, John is reading books.
	* Every week, John is reading books.
	d. Yesterday afternoon, John was reading books.
	* Tomorrow afternoon, John was reading books.

* indicates that the sentence is incorrect.

Steinhauer and Ullman [12] used these paradigms to present the sentences exemplified in (5) to English native speakers. The past tense of the verb is in agreement with the past time in the temporal sense (5a), but the present time of the verb and the past time causes temporal violation (5b) in the temporal sense. The researchers found that tense violation elicited a left anterior negativity (LAN) of 300–500 ms after the onset of the verb, followed by centro-posterior positivity (P600) in the 600–900 ms time-range. Steinhauer and Ullman [12] interpreted the LAN-P600 pattern as indicating the morphosyntactic processing of tense violation. In a later similar study, Newman et al. [21] reported that the LAN-P600 biphasic effect is produced by verbs with regular past tense, while only the P600 effect is produced by verbs with irregular past tense. Findings from Baggio’s [22] study of Dutch tenses (see (6) for experimental materials) showed an LAN-P600 biphasic effect, but that LAN amplitude is larger, while P600 amplitude is smaller. Baggio [22] argued that LAN is involved in semantic processing, and proposed a complete syntactic analysis and understanding model, in which the time reference calculation is entrusted to semantic processing, as the primary stage of syntactic structure construction.

**Table:** 

(5)	a. Yesterday, I sailed Diane’s boat to Boston.
	b. * Yesterday, I sail Diane’s boat to Boston.
(6)	a. Afgelopen zondag lakte Vincent de kozijnen van zijn landhuis.
	Last Sunday Vincent painted the window frames of his country house.
	b. * Afgelopen zondag lakt Vincent de kozijnen van zijn landhuis.
	* Last Sunday Vincent paints the window frames of his country house.

* indicates that the sentence is incorrect.

Fonteneau et al. [13] used the violation paradigm to study aspect violation processing in French (the experimental material is exemplified in (7)). They found that aspect violation sentences elicit a frontal positivity combined with a posterior negativity effect in the 457–556 ms time window after the verb onset, and these two components are different from typical language processing ERP components such as N400, LAN and P600. In our study, we also found that the present progressive tense can encode not only temporal information, but also aspectual information.

**Table:** 

(7)	* Demain l’étudiant lisait le livre.
	* Tomorrow the student was reading the book.

* indicates that the sentence is incorrect.

Zhang and Zhang [14] used the violation paradigm to study the grammatical aspect in Chinese (see (8) for experimental materials), and found that the aspect violation caused by the disagreement between temporal adverbs and aspect markers elicited 200–400 ms posterior and left central negativity, followed by a P600. The early posterior and left central negativity is different from N400 and LAN. This is due to aspect marker mismatching or aspect errors, and the processing of grammatical aspect is not by semantics alone [14]. Similarly, Qiu and Zhou [15] studied the aspect violation phenomenon in Chinese temporal adverbs and the aspect marker *guo* (see (9) for experimental materials), and found that disagreement between temporal adverbs and aspect markers elicited a centro-parietal P600 effect.

**Table:** 

(8)	* 苏君 正在 预备 了 水果 和 甜点。
	* Sujun zhengzai yubei *le* shuiguo he tiandian
	* Sujun zhengzai prepare-*le* fruits and dessert
	* Sujun zhengzai (PROG, ‘ongoing’) prepare *le* (PERF) fruit and cookies.
(9)	* 下个月 联合国 派出过 特别 调查组。
	* Xiageyue lianheguo paichu *guo* tebie diaochazu
	* Next month UN dispatch-*guo* special investigation unit
	* Next month The United Nations dispatched a special investigation unit.

* indicates that the sentence is incorrect.

Monique et al. [23] used the aspect violation paradigm (as shown in (10)) to explore whether grammatical aspect processing is similar to semantic and morphosyntactic processing. The results showed that semantic violation elicited an N400 effect, morphosyntactic violation elicited a P600 effect, and aspect violation elicited early negativity from 250 to 350 ms, followed by a P600 with less amplitude. These differences indicate that aspect processing is not purely semantic processing or morphological syntactic processing. In addition, Monique et al. [23] argued that the early negativity was due to prediction error. When the prediction of aspectual information was in disagreement with the temporal context, early negativity would be elicited. Similar results were found in a Japanese study by Masatak [24], which showed that an early anterior negative was elicited only when there was sufficient time for prediction.

**Table:** 

(10)	* Every Tuesday, John is swimming in the pool.

* indicates that the sentence is incorrect.

Jan and Christina [25] studied the grammatical aspect in Russian by using the violation paradigm (as shown in (11)), and native Russian speakers as the participants. They found that aspect violation sentence processing elicited a late P600. These results suggested that aspect violation processing in Russian is similar to that of morphosyntactic violation, which is consistent with the interpretation of aspect as a typical syntactic category.

**Table:** 

(11)	* Každyj četverg torgovec napilsjapf v pivnoj.
	* Every Thursday the merchant got drunk in the pub.

* indicates that the sentence is incorrect.

However, in addition to the P600 effect, aspectual processing can also induce late AN. The late AN effect may lead to a reinterpretation process of aspect [26]. Paczynski et al. [27] studied the phenomenon of English aspectual coercion, and found a continuous negativity in the late time course of 500–1200 ms. This was due to syntactic structure repair. Similarly, Masatak [24] also observed a late AN, and the results showed that it reflected the reinterpretation process of aspect.

To summarize, research into aspectual processing has gone in several directions. P600 has been found in the aspect processing of English, Chinese and Russian, indicating that both Indo-European languages’ aspect processing and Chinese’s aspect processing is correlated with syntactic processing. However, French aspectual processing showed a frontal positivity combined with a posterior negativity effect; English aspectual processing showed LAN; Chinese aspectual processing showed early posterior and left central negativity. Additionally, both early and late AN were observed in aspectual processing.

### 1.2. The Present Study

The primary means of expressing grammatical category in Chinese are word order and function words. Moreover, Chinese lacks grammatical forms for expressing syntactic category, such as number, gender and tense [28]. In the tense aspect category, Chinese verbs have no morphological markers of tense, but they can use time adverbs, time nouns, a few aspect markers and their combinations to express time information [29]. The combination of verbs with different lexical and grammatical aspects is a common way to express aspect in language. However, the category of aspect can be expressed by verb inflection, and the lexical aspect of verbs cannot be separated from the grammatical aspect. For example, the accomplishment verb “write” and the progressive aspect suffix “-ing” are combined with “be” to form the progressive aspect; “write” and the suffix “-ed” of perfectives are combined with have/has to form perfective aspect. In Chinese, the collocation of verbs with different lexical aspect and aspect markers is also an important means of expressing Chinese aspect. An example of the collocation of verbs with different lexical aspect and aspect markers is shown in (12). It demonstrates that the lexical aspect in verbs and aspect markers are independent of each other.

**Table:** 

(12)	a. 苏珊 收到 了 礼物。
	Susan shoudao *le* liwu
	Susan receive-*le* liwu
	Susan received the present.
	b. * 苏珊 收到 着 礼物。
	* Susan shoudao *zhe* liwu
	* Susan receive-*zhe* liwu
	* Susan is receiving the present.

* indicates that the sentence is incorrect.

Most research on the lexical aspect of verbs has been based on [10] quartering method. Summative, instantaneity and consequentiality are the semantic features most similar to perfectives. Therefore, the achievement verbs are the most typical lexical expression of perfectives, while the state verbs have the lowest combining ability with perfectives [30]. In addition, the usage of verbs with different lexical aspect varies across tense and form. The likelihood of using the general past tense of the achievement verbs and the accomplishment verbs is higher than that of the state verbs or the activity verbs would be in the same situation [31]. At all levels, inflectional changes and lexical types are dependent: the progressive “-ing” is used with activity verbs, and the perfective aspect is used with instantaneous verbs [8]. The lexical aspect of verbs in Chinese also follows Vendler’s [10] and Smith’s [7] classification, as shown in Table 2. The verbs of the four types of lexical aspect can constitute the aspect of a sentence with the corresponding aspect markers.

Chinese has a rich aspect system, with several aspect markers with their own meanings and functions [32]. The auxiliary words *guo*, *le* and *zhe* attached to verbs are usually used as aspect markers to indicate the completion and progress of actions [29,33]. The temporal system of Chinese is more semantically complex than that of tense markers in Indo-European languages [22]. They primarily encode aspectual information, and their temporal interpretation also depends on sentence context and discourse principles. Chang [34] investigated the distribution rules of the syntactic features of *zai*, *le*, *zhe* and *guo* in the four main lexical types. Chinese sentences’ temporal information is often determined by the lexical type verbs and aspect markers. The state verbs and *zhe* typically represent the current time; achievement verbs with *le* or *guo* typically express the past time; if the accomplishment verb is used with *zai* to express the meaning of process, it means the present time. The activity verbs usually mean the present time in its unmarked form (i.e., habitual active sentences) or when they are used with the progressive markers *zai* and *zhe* [34]. The aspect marker *le* also has a corresponding relationship with the lexical verb types, and *le* often follows the accomplishment verbs to indicate an action’s completion [35]. In sum, the combination of verbs with varying lexical aspects and aspect markers affects sentences’ aspects.

In conclusion, previous aspect processing research has focused on the disagreement between temporal nouns/temporal adverbs and aspect information. The participants predict the ensuing verbs’ aspect information through the temporal information expressed by temporal noun/temporal adverbs. However, little attention has been paid to the verb’s intrinsic temporal features’ matching processing (i.e., verbs’ lexical aspect) and aspect information, that is, how the verb’s lexical aspect’s information predicts the following grammatical aspect information in sentences’ processing. This may be due to the means of expressing aspect information in Indo-European languages, and it is difficult to separate the verbs’ lexical aspect information from their grammatical aspect information. Chinese verbs lack morphological changes, and their grammatical aspect information is expressed by aspect markers which are independent of verbs in form. This made it possible for us to study the processing of the combination of verbs’ lexical aspect and grammatical aspect information. This study explores how the collocation of various lexical aspects of Chinese verbs and aspect markers affects the information processing of Chinese sentences’ aspect. We explored the ERP components elicited by the combination of various lexical aspects of verbs and aspect markers. There were no time nouns or time adverbs in the sentences in this experiment, only verbs with varying lexical aspects and aspect markers. The verbs in aspect violation sentences were achievement verbs that can be combined with le, while the verbs with this kind of lexical aspect cannot be combined with *zhe*. According to the design of this study and previous research, we had the following expectations for the research results. First, Chinese aspect processing would only involve the syntactic level, and the P600 would be involved in syntactic processing. This has been reported in most previous studies; second, because the aspect marker is next to the verb, it may lead to instant prediction of the upcoming aspect marker when the participants see verbs with different lexical types. Therefore, violating the combination of aspect markers and lexical verb types may lead to components other than P600 affecting syntactic processing. In addition, Jiang et al. [36] and Qiu and Zhou et al. [22,37] found that the post-critical words induced a sustained negative effect, which also appeared in violation sentences’ sentence-final words [19,38]. In the design of this study, there is only one word after the critical words, so it is also the sentence-final word. Therefore, we expected to find a similar sustained negativity for the post-critical word.

## 2. Materials and Methods

### 2.1. Participants

In this study, we selected 22 native Chinese students aged 18–24 years as participants, 2 participants were excluded from the ERP analysis due to excessive artifacts. All participants were classified as right-handed based on the Edinburgh handedness inventory [39], and all had normal or corrected-to-normal vision. The participants had no history of reading disability or neurological disease. We obtained informed consent from all participants prior to the experiment, and they were paid for their participation.

This research was approved by the ethics committee of School of Medicine of Huaqiao University (ethic code: M2021014).

### 2.2. Stimuli

The stimuli consisted of correct sentences and violation sentences constituted by lexical verb types and aspect marks (Table 3).

In the stimulus materials we designed, the lexical aspect and the grammatical aspect of verbs were independent of each other. The lexical aspect of verbs is only reflected in the verb, and the grammatical aspect is only reflected in the aspect marker. “Anna/xiang/*zhe*/Beijing” is a correct sentence, in which “xiang” is a state verb with the semantic feature of (−punctual). Thus, it is correctly combined with the progressive marker *zhe*. Meanwhile, “*Anna/dao/*zhe*/Beijing” is an aspect violation sentence, in which “dao” is an accomplishment verb with the semantic feature of (+punctual). Thus, it is incorrect to use it with the progressive marker *zhe*.

We selected the experimental materials from common words in the HSK (the Hanyu Shuiping Kaoshi (HSK) is a standardized international Chinese proficiency test for non-Chinese native speakers (including foreigners, overseas Chinese, Chinese Americans and Chinese ethnic minorities). Level 6 is the highest HSK level in the new Chinese proficiency test. Candidates who pass HSK level 6 can easily understand Chinese information they hear or read, and express their opinions fluently in Chinese, in both oral and written form) vocabulary. The legitimacy of verb combinations with different lexical types and aspect markers refers to Su et al. [40]. As there is no previous experimental paradigm for reference in this experiment, we selected 34 native Chinese college students to judge the correctness of 66 sentences before determining the key experimental materials and compiling the filling materials. There were five options after each sentence, which corresponded to the five-level scale. They were: totally acceptable (5 points), generally acceptable (4 points), basically acceptable (3 points), generally unacceptable (2 points) and totally unacceptable (1 point). The higher the sentence’s score, the more feasible the sentence. Post-hoc Newman–Keuls analysis of variance showed that the average score of sentences with a correct condition was 4.1, and that of sentences with an aspect violation was 2.8. Sentences with aspect violation were less acceptable than those with correct conditions (*p* < 0.01). After completing the steps above, we could modify and compile the experimental materials accordingly.

Experimental materials included key materials and corresponding amounts of filler stimuli. The sentences in the key materials were divided into correct sentences and sentences with aspect violations, with a total of 60 sentences. The correct sentences were sentences with the right collocation of verbs and *zhe*, while the sentences with aspect violation were sentences with the wrong collocation of verbs and *zhe*. Filler stimuli included 30 semantic violation sentences with *le* and 30 correct sentences with *zhe*, for a total of 60.

We dealt with the key materials in Latin square to offset the inertia of the verbs in the key materials and balance the inertia of the correct materials and the wrong materials, so as to prevent the participants from predicting the aspect markers after the verbs. In order to match the key materials’ conditions, we created two lists. Each participant only read one list, and there were 90 sentences in each (60 key materials + 30 filler stimuli). We used the aspect marker *zhe* as the critical word in each sentence, and the noun before the period as the sentence-final word. We wrote the experimental materials in E-prime 3.0.

### 2.3. Procedure

We presented the sentences on the screen one-by-one and broken into fragments. The practice and formal experiment were expected to last about 25 min. We presented the experimental materials in four fragments, for example 安娜/想/着/北京. Before the experiment, we told the participants to minimize their body movement and pay attention. At the beginning of the experiment, instructions in black script and a white background appeared on the screen. Then, participants were shown nine practice sentences to familiarize them with the experiment’s procedures. After practicing, they could choose to either return to practice or start the formal experiment.

At the beginning of the formal experiment, the center of the screen first presented a “+” for 80 ms to remind the participants to start the experiment. Then there was an empty screen for 600 ms, and the sentences were presented word-by-word on the screen in the form of lexical fragments. Each lexical fragment’s presentation time was 600 ms, and there was a 400 ms interval between every two lexical fragments. Then an 800 ms empty screen appeared, followed by a 3000 ms “???”. When “???” appeared, the participants had to judge whether the sentence was acceptable. If so, they were to press “D” on the keyboard, and if the sentence was wrong, they were to press “K”.

### 2.4. Electrophysiological Recording

The electrode cap used in the experiment had 64 poles, and we selected 15 electrodes as the observation electrodes: F5, FZ, F6, FC5, FCZ, FC6, C5, CZ, C6, CP5, CPZ, CP6, P5, PZ and P6. We placed additional electrodes below and above the left eye and at the corners of both eyes, to monitor the eyes’ vertical and horizontal movement. We selected M1 and M2 electrodes as reference electrodes, and all electrodes’ impedances were kept at 5 KΩ thereafter.

### 2.5. Electrophysiological Data Analysis

In the experiment, we used Neuroscan EEG recording equipment to collect the data, Curry8 to preprocess the data, MATLAB and Origin to sort the topographical isovoltage map and waveform diagram, and R for statistical analysis. Large artefacts (exceeding ± 100 μV) were automatically removed from the analysis, and we filtered the collected EEGs with a 30 Hz low-pass filter. The baseline was set to 200 ms (the critical words) and 100 ms (the sentence-final words) prior to the onset of the third fragment (aspect markers). There were two time windows for critical word observation: 300–500 ms and 500–700 ms; the post-critical words’ observation window was 500–700 ms.

We analyzed the experimental data from the midline and bilateral parts of the scalp. We selected three electrodes in the midline: FZ, CZ and PZ. Bilateral brain regions included two topographic dimensions: region (frontal, fronto-central, central, centro-parietal and parietal) and hemisphere (left, medial, right). These regions crisscrossed with the hemispheres to form 15 regions of interest with three electrodes each: left frontal (F3, F5, F7), left fronto-central (FC3, FC5, FT7), left central (C3, C5, T7), left centro-parietal (CP3, CP5,TP7), left parietal (P3, P5, P7), medial frontal (F1, FZ, F2), medial fronto-central (FC1, FCZ, FC2), medial central (C1, CZ, C2), medial centro-parietal (CP1, CPZ, CP2), medial parietal (P1, PZ, P2), right frontal (F4, F6, F8), right fronto-central (FC4, FC6, FT8), right central (C4, C6, T8), right centro-parietal (CP4, CP6, TP8) and right parietal (P4, P6, P8). The midline analysis was the mixed-effects modelling analysis of two factors: Mixed model = M.01 <- lmer (Avgamp~Point*Condition + (1|subject), data = x), where Point was the electrodes, Condition was the experimental conditions and Avgamp was the average amplitude.

Bilateral analysis was the mixed-effects modelling analysis of three factors: Mixed model = M.01 <- lmer (Avgamp~Hemi*Area*Condition + (1|subject), data = x), in which Hemi represented hemispheres, Area represented regions, Condition represented experimental conditions and Avgamp represented the average amplitude. All factors were of interest, and we analyzed their main effects with the anova () function. We compared them post hoc with the glht () function. Finally, we reported the *p*-values.

## 3. Results

### 3.1. Behavioral Data

For Chinese native speakers, the aspect violation sentences’ accuracy was 93.8%, and the correct sentences’ accuracy was 97.1%. After mixed-effects modelling analysis with R, we found that the main effect of condition was significant (*χ*^2^(1) = 5.783, *p* < 0.001). This indicated that participants were less accurate on aspect violation sentences.

### 3.2. Electrophysiological Data

#### 3.2.1. Critical Words

**Figure 1:**
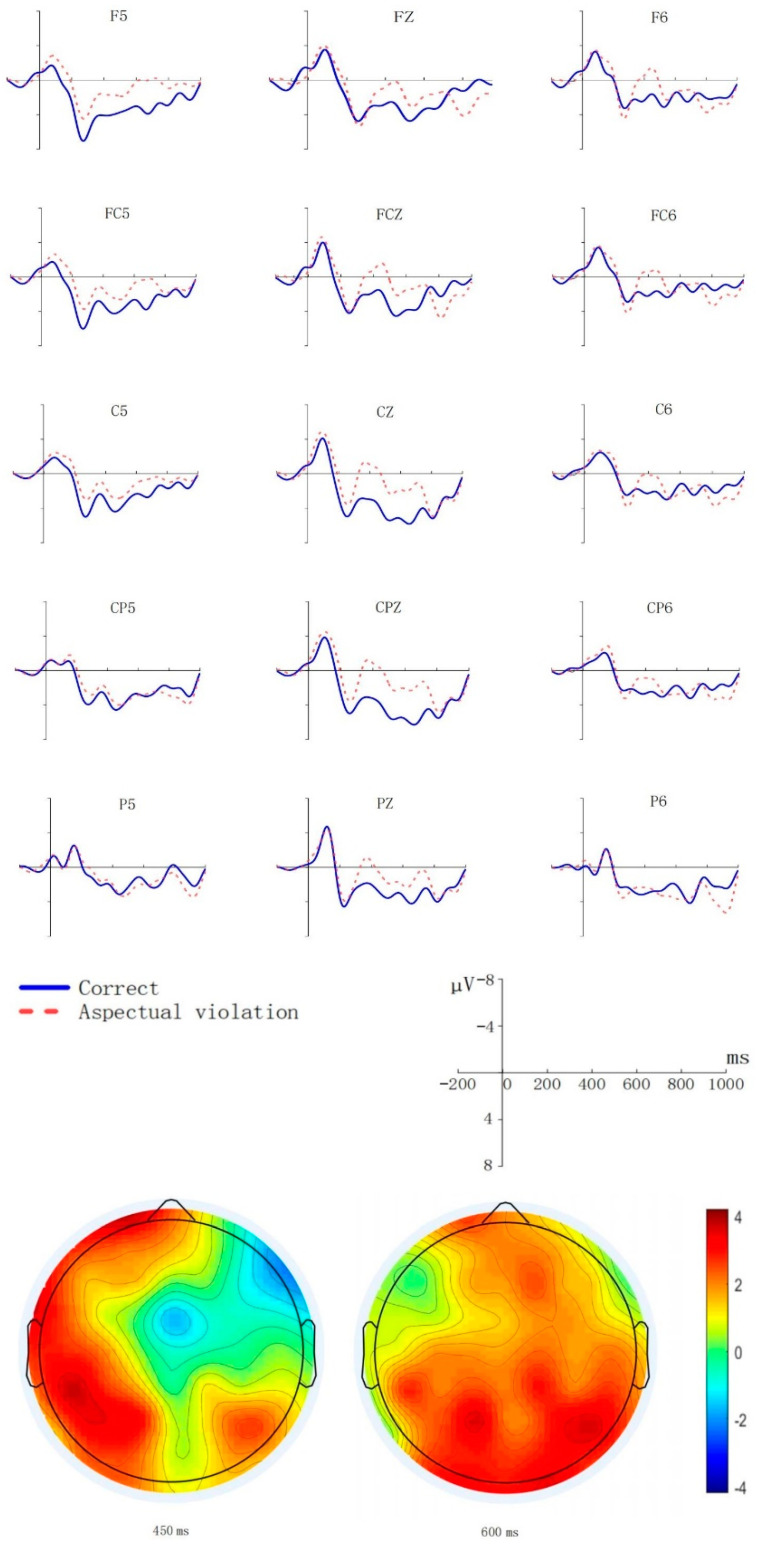
Grand average (*N* = 20, with 20 valid samples) ERPs at 15 exemplar electrodes time-locked to the onset (0 ms) of the aspect markers as a function of aspect agreement. The *X*-axis represents the duration, and each hash mark represents 200 ms. The *Y*-axis represents the voltage, which ranged from −8 to +8 µV. Negativity is plotted upward. The agreement effect’s scalp distribution in the early AN and P600 time windows is also depicted in the topographic maps. The topographical isovoltage map represents the aspect violation effects at 450 ms and 600 ms.

We used R to analyze and process all of the data, and used mixed-effects modelling to fit the participants’ average amplitude. We reported the *β* values as the statistical results’ effect quality. Figure 1 shows waveforms of aspect violation sentences and correct sentences and scalp distribution of aspect violation processing in the N400-like and P600 time window.

- Midline

We used the lmer function from the lme4 package to analyze the midline average amplitude. The mixed-effects modelling after fitting is as follows:

Mixed model = M.01 <- lmer (Avgamp~Point*Condition + (1|subject), data = x). In this model, Avgamp was the participants’ average amplitude and was the experiment’s dependent variable. Independent variable 1 was the electrodes, and independent variable 2 was the condition. The electrodes and conditions were the fixed-effects of the mixed-effects modelling, and participants were the random-effects of the mixed-effects modelling. There were three levels of electrodes—FZ, CZ and PZ; there were two levels of condition: the correct sentence and the aspect violation sentence.

The 300–500 ms time window—The mixed-effects modelling’s fitting results showed that the condition’s main effect was significant (*χ*^2^(1) = 5.578, *p* = 0.018), and post hoc comparison suggested that the average amplitude caused by aspect violation sentences was more negative than that caused by correct sentences (*β* = −1.08, SE = 0.47, *z* = −2.32, *p* = 0.021). The main effect of the electrodes was not significant (*χ*^2^(2) = 1.20, *p* = 0.549). The interaction between the electrode and the condition was significant (*χ*^2^(2) = 6.20, *p* = 0.045), and post hoc comparison suggested that the average amplitude caused by aspect violation sentences was more negative than that caused by correct sentences at CZ (*β* = −2.65, SE = 0.54, *z* = −4.95, *p* < 0.001); at FZ and PZ, there was no statistically significant difference in the average amplitude caused by aspect violation sentences and correct sentences.

The 500–700 ms time window—The mixed-effects modelling’s fitting results showed that the main effect of the condition was marginally significant (*χ*^2^(2) = 3.80, *p* = 0.051), and post hoc comparison suggested that the average amplitude caused by aspect violation sentences and by correct sentences was marginally significant (*β* = −1.27, SE = 0.66, *z* = −1.94, *p* = 0.053). The main effect of the electrodes was not significant (*χ*^2^(2) = 3.45, *p* = 0.178). The interaction between the electrode and the condition was also not significant (*χ*^2^(2) = 1.83, *p* = 0.401).

- Lateral

We used the lme4 package’s lmer function to analyze the midline average amplitudes. The mixed-effects modelling after fitting was as follows:

Mixed model = M.01 <- lmer (Avgamp~Hemi*Area*Condition + (1|subject), data = x). In this model, Avgamp is the participants’ average amplitude and the experiment’s dependent variable. Independent variable 1 is the hemisphere, independent variable 2 is the region and independent variable 3 is the condition. The hemisphere, the region and the condition were the fixed-effects in the mixed-effects modelling, participants were the random-effects in the mixed-effects modelling. There are three hemispheres: left, medial and right; five regions: frontal, fronto-central, central, centro-parietal and parietal; and two conditions: the correct sentence and the aspect violation sentence.

The 300–500 ms time window—The mixed-effects modelling’s fitting results showed that the condition’s main effect was not significant (*χ*^2^(1) = 2.26, *p* = 0.133); the hemisphere’s main effect was significant (*χ*^2^(2) = 10.02, *p* = 0.007), and post hoc comparison suggested that the average amplitude in the medial is more negative than that in the left hemisphere (*β* = −1.35, SE = 0.35, *z* = −3.82, *p* < 0.001) and the average amplitude in the right hemisphere is more negative than that in the left hemisphere (*β* = −1.03, SE = 0.35, *z* = 2.90, *p* = 0.010); the left hemisphere’s average amplitude was 0.888 μV, the medial was 0.318 μV and −0.313 μV in the right hemisphere. The region’s main effect was not significant (*χ*^2^(4) = 4.32, *p* = 0.365). None of the interaction effects were statistically significant.

The 500–700 ms time window—The mixed-effects modelling’s fitting results showed that the condition’s main effect was not significant (*χ*^2^(1) = 0.11, *p* = 0.138). The main effect of the hemisphere was significant (*χ*^2^(2) = 20.29, *p* < 0.001), and post hoc comparison suggested that the average amplitude in the medial is more positive than that in the left hemisphere (*β* = −1.61, SE = 0.36, *z* = −4.42, *p* < 0.001) and the average amplitude in the right hemisphere is more positive than that in the left hemisphere (*β* = 1.05, SE = 0.36, *z* = 2.89, *p* = 0.011); the average amplitude of the left hemisphere was −0.554 μV, the medial was 1.056 μV and 0.498 μV in the right hemisphere. The region’s main effect was significant (*χ*^2^(4) = 14.69, *p* = 0.005), and post hoc comparison suggested that the average amplitude in the centro-parietal region is more positive than that in the fronto-central region (*β* = 1.44, SE = 0.47, *z* = 3.05, *p* = 0.020) and the average amplitude in the parietal region is more positive than that in the fronto-central region (*β* = 1.32, SE = 0.47, *z* = 2.78, *p* = 0.043); the average frontal amplitude was −0.169 μV, the fronto-central was −0.500 μV, the central was 0.571 μV; the centro-parietal was 0.945 μV and the parietal was 0.820 μV. The interaction between the hemisphere and the condition was marginally significant (*χ*^2^(2) = 5.30, *p* = 0.071) and post hoc comparison suggested that the average amplitude caused by aspect violation sentences and by correct sentences was marginally significant at the right hemisphere (*β* = 0.74, SE = 0.46, *z* = 1.61, *p* = 0.091); the interaction between the region and the condition was significant (*χ*^2^(4) = 8.62, *p* = 0.036) and post hoc comparison suggested that the average amplitude caused by aspect violation sentences exceeded that caused by correct sentences in the parietal region (*β* = 9.71, SE = 1.15, *z* = 2.62, *p* = 0.024), and in the centro-parietal region, the average amplitude caused by aspect violation sentences and by correct sentences was marginally significant (*β* = 8.11, SE = 1.15, *z* = 2.84, *p* = 0.056). The 3-way interaction was not significant (*χ*^2^(8) = 3.78, *p* = 0.876).

According to critical words’ statistical analysis above, the results are consistent with an N400-like in the early time window and P600 at the late time window. Additionally, the waveforms of aspect violation sentences and correct sentences and scalp distribution of aspect violation processing also support the statistical results.

#### 3.2.2. Post-Critical Words

**Figure 2:**
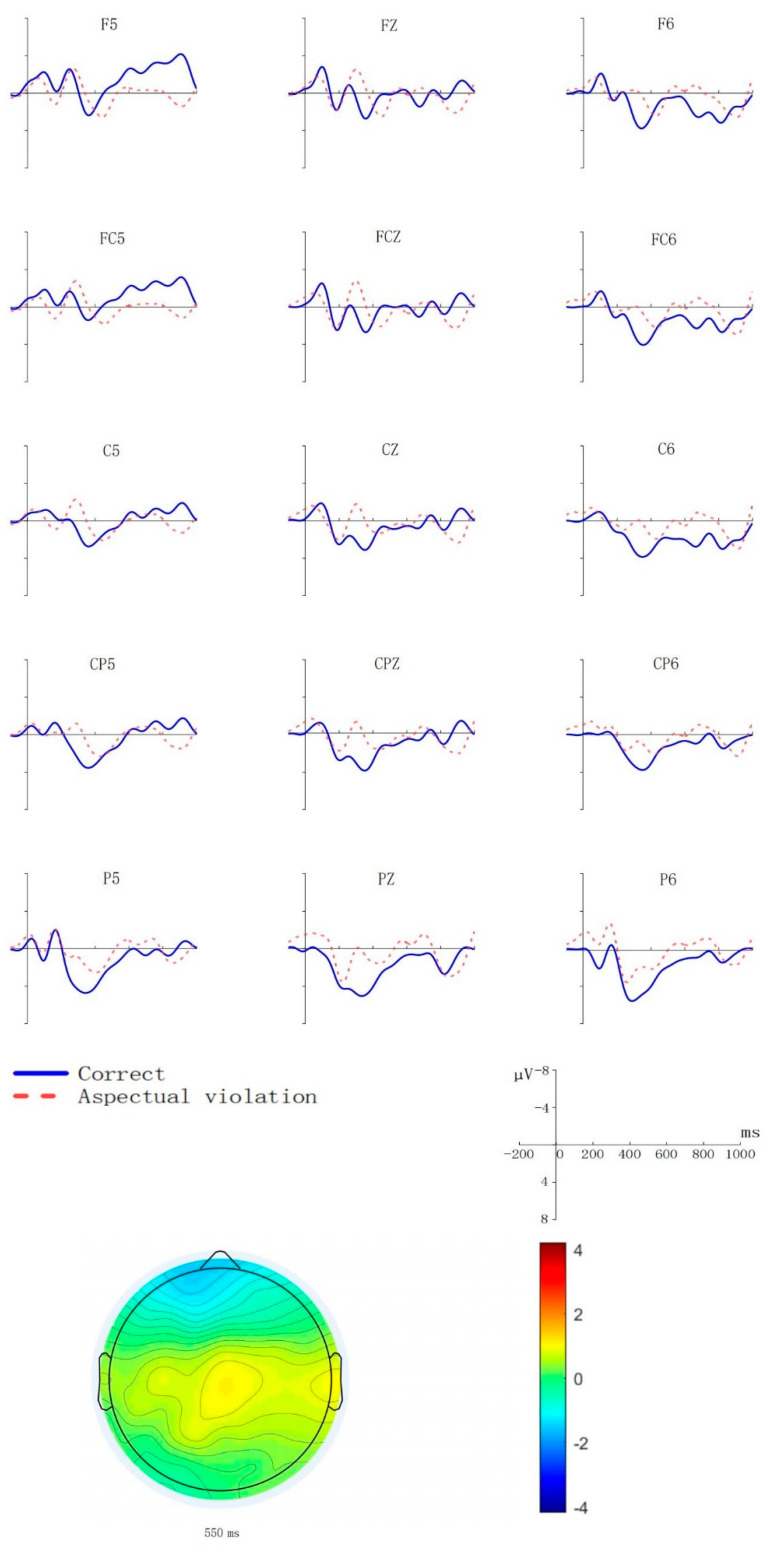
Grand average (*N* = 20, with 20 valid samples) ERPs at 15 exemplar electrodes time-locked to the onset (0 ms) of aspect markers as a function of aspect agreement. The *X*-axis represents the duration, and each hash mark represents 200 ms. The *Y*-axis represents the voltage, which ranged from −8 to +8 µV. Negativity is plotted upward. The agreement effect’s scalp distribution in the late AN time window is also depicted in the topographic map. The topographical isovoltage map represents the aspect violation effects at 550 ms.

Figure 2 shows waveforms of aspect violation sentences and correct sentences and scalp distribution of aspect violation processing in the late AN time window.

- Midline

We used the lme4 package’s lmer function to analyze the midline average amplitude. The mixed-effects modelling after fitting is as follows:

Mixed model = M.01 <- lmer (Avgamp~Point*Condition + (1|subject), data = x). In this model, Avgamp was the participants’ average amplitude which was the experiment’s dependent variable. Independent variable 1 was the electrodes, and independent variable 2 was the condition. The electrodes and conditions were the fixed-effects of the mixed-effects modelling; participants were the mixed-effects modelling’s random effects. There are three electrodes—FZ, CZ and PZ, and two conditions: the correct sentence and the aspect violation sentence.

The 500–700 ms time window—The mixed-effects modelling’s fitting results showed that the main effect of the condition was not significant (*χ*^2^(1) = 0.60, *p* = 0.440), and the main effect of the electrodes was not significant (*χ*^2^(2) = 2.05, *p* = 0.359). The interaction between the electrode and the condition was not significant (*χ*^2^(2) = 3.73, *p* = 0.155).

- Lateral

We used the lme4 package’s lmer function to analyze the midline average amplitude. The mixed-effects modelling after fitting is as follows:

Mixed model = M.01 <- lmer (Avgamp~Hemi*Area*Condition + (1|subject), data = x). In this model, Avgamp was the participants’ average amplitude and the experiment’s dependent variable. Independent variable 1 was the hemisphere, independent variable 2 was the region and independent variable 3 was the condition. The hemisphere, the region and the condition were the mixed-effects modelling’s fixed-effects, and participants were the mixed-effects modelling’s random-effects. The hemispheres have three levels—left, medial and right; the regions have five levels—frontal, fronto-central, central, centro-parietal and parietal; condition has two levels—the correct sentence and the aspect violation sentence.

The 500–700 ms time window—The mixed-effects modelling’s fitting results showed that the condition’s main effect was marginally significant (*χ*^2^(1) = 2.96, *p* = 0.086) and post hoc comparison suggested that the average amplitude caused by aspect violation sentences and by correct sentences was marginally significant (*β* = 0.36, SE = 0.22, *z* = 1.66, *p* = 0.097). The hemisphere’s main effect was significant (*χ*^2^(2) = 31.53, *p* < 0.001), and post hoc comparison suggested that the average amplitude in the left hemisphere is more negative than that in the medial (*β* = −0.68, SE = 0.26, *z* = −2.60, *p* = 0.025), the average amplitude in the medial is more negative than that in the right hemisphere (*β* = 0.65, SE = 0.26, *z* = 2.48, *p* = 0.036) and the average amplitude in the left hemisphere is more negative than that in the right hemisphere (*β* = 1.33, SE = 0.26, *z* = 5.08, *p* < 0.001); the left hemisphere’s average amplitude was −0.723 μV, the medial was −0.272 μV and 0.766 μV in the right hemisphere. The region’s main effect was significant (*χ*^2^(4) = 10.24, *p* = 0.037), and post hoc comparison suggested that the average amplitude in the fronto-central region is more negative than that in the parietal region (*β* = 0.86, SE = 0.34, *z* = 2.51, *p* = 0.028); the frontal’s average amplitude was −0.213 μV; the fronto-central was −0.650 μV; the central was −0.047 μV; the centro-parietal was 0.108 μV; and the parietal was 0.422 μV. The interaction between the hemisphere and the condition was significant (*χ*^2^(2) = 7.28, *p* = 0.026) and post hoc comparison suggested that the average amplitude caused by aspect violation sentences exceeded that caused by correct sentences at the left hemisphere (*β* = 0.78, SE = 0.34, *z* = 2.30, *p* = 0.022). The interaction between region and condition was significant (*χ*^2^(4) = 12.58, *p* = 0.014) and post hoc comparison suggested that the average amplitude caused by aspect violation sentences surpassed that caused by correct sentences in the frontal region, (*β* = −1.13, SE = 0.53, *z* = −2.12, *p* = 0.034) and in the central region, the average amplitude caused by aspect violation sentences exceeded that caused by correct sentences (*β* = 0.98, SE = 0.34, *z* = 2.92, *p* = 0.003),and in the centro-parietal region, the average amplitude caused by aspect violation sentences and that caused by correct sentences was marginally significant (*β* = 0.80, SE = 0.42, *z* = 1.90, *p* = 0.057). The 3-way interaction was not significant (*χ*^2^(8) = 1.21, *p* = 0.997).

According to critical words’ statistical analysis, the results are consistent with an AN in the late time window. Additionally, the waveforms of aspect violation sentences and correct sentences and scalp distribution of aspect violation processing also support the statistical results. This is consistent with predictions.

## 4. Discussion

We studied the cognitive collocation processing of the lexical aspect of verbs and aspect markers. During processing, matching achievement verbs with the progressive marker *zhe* elicited the corresponding ERPs components. Based on previous research results and the experimental results of this study, we believe that the mismatch between the lexical aspect of verbs and aspect markers may involve semantic processing, syntactic processing and repair processes. The ERPs’ experimental results confirmed this hypothesis. Unlike the correct sentences, in the early time window, the critical words in aspect violation sentences elicited N400-like. Then, they produced a positive effect (P600) in the posterior region while the aspect violation sentences’ post-critical words elicited a late anterior negative (late AN).

### 4.1. Critical Words (Aspectual Markers)

When a verb appeared, so did its lexical types, and the participants could predict the aspect markers according to them. According to the distribution of topographical map in 300–500 ms time window in Figure 1, there was an early negative which is distributed in the centro-right region of the brain, and a prominent negativity peak in the corresponding time window. This was different from the conclusions of Zhang and Zhang [14] and Qiu and Zhou [15] who did not find that aspect markers elicited the N400-like. According to Masatak [24], only when the participants made a prediction that was wrong, would the early AN but not N400-like occur in 300–500 ms time window. In the present study, the N400-like indicated that the participants had a process of semantic processing. When the verb appeared, the participants would predict which aspect marker would appear later. As the lexical aspect of verbs is related to the intrinsic semantic feature of the verb or verb phrase [8,9], when the wrong aspect marker appeared, it will cause semantic mismatch. The verb with the semantic feature (+punctual) triggered the prediction of the aspect marker *le*, but the final one was the aspect marker *zhe*, which made a semantic mismatch. Therefore, the N400-like was elicited.

Subsequently, the aspect violation sentence elicited a clearer positive effect that was distributed throughout the posterior area, and a prominent positivity peak in the corresponding time window. In addition, aspect markers are involved in syntax, so we could determine that the positive effect was P600. This finding was consistent with that of Zhang and Zhang [14], Qiu and Zhou [15], Monique et al. [23] and Jan and Christina [25]. They all found P600 in their research, which corresponded to the explanation of aspect as a typical syntactic category. P600 is an ERP component used for syntactic repair or reanalysis [41]. P600 embodies the unified connection established by the syntactic framework, and it is influenced by either syntactic ambiguity or complexity [42]. When the aspect marker predictions were wrong, the brain needed to repair the incorrect aspect information. Therefore, P600 appeared. P600 also reflected the semantic coordination process at different syntactic levels [43]. Qiu and Zhou [15] showed that during sentence processing, lexical items must be combined into a legal syntactic structure and coherent semantic characterization. In the present study, when the lexical aspect of verbs and aspect markers did not match, the mismatch of temporal semantic features in the lexical aspect of verbs and aspect markers led to syntactic structure repair. This elicited P600. This result is consistent with that of Zhang et al. [44] and Qiu and Zhou [15], but the experimental materials in the present study were different from those of Zhang et al. [44] and Qiu and Zhou [15]. We embedded the aspect marker *zhe* between the predicate verb and the object noun; Zhang et al. [44] inserted an adjective between the predicate verb and the object noun; Qiu and Zhou [15] inserted the aspect marker *guo* between the predicate verb and the object noun, but added a time adverb preceding the sentence. In Chinese, the combination of the aspect marker and the lexical aspect of verbs is one means of aspect expression. Verbs have some semantic features, such as (±dynamic), (±punctual) and (±telic) which correspond to the verb’s lexical aspect. Therefore, in sentence processing, the mismatch between the lexical aspect of verbs and aspect markers leads to time semantics disagreement in the syntactic structure, resulting in the P600 effect.

### 4.2. Post-Critical Words

During cognitive processing, the effect of the critical words can extend to the post-critical words. This is why we analyzed the post-critical words. Jiang et al. [36] and Qiu and Zhou et al. [15,37] studied post-critical words, and found a sustained negative effect, which reflected the secondary repair process. Different from the above studies, in our study, the post-critical words were the words after the aspect markers, and we found the following component according to the waveforms and topographic map.

In the 500–700 ms time window, the aspect violation sentences elicited a late negative effect which distributed anteriorly, namely, late AN. These results were consistent with the findings of Baggio et al. [38], Bott [26], Paczynski [27], Wittenberg et al. [45] and Masatak [24]. Baggio et al. [38] found a sustained negativity in the frontal distribution which began 500 ms after the onset of the verb. Bott [26] reported that a sustained anterior negative effect was elicited 500 ms after the onset of critical verbs. Paczynski [27] found a sustained negativity from 500 to 1200 ms after the onset of the verb. Wittenberg et al. [45] observed that there was a sustained, anterior negative effect 500 ms after the onset of the verb. Masatak [24] also found an anterior negativity in the 500–700 ms time window. All of the above researchers observed the late AN, and thought that the negative effect was due to the aspect reinterpretation process, because aspect reinterpretation of aspect was necessary to solving the aspect mismatch. In the present study, the late AN appeared in the post-critical word, which may be aspects’ secondary interpretation process. Because P600 in the critical word had corrected the aspect information, when the post-critical word appeared, it may have reflected the aspect’s secondary repair process.

In the present study, the post-critical word was also the sentence-final word. Baggio [22], Baggio et al. [38], Hagoort [46], Hagoort et al. [47] and Qiu and Zhou [15] found a sustained negative effect on the sentence-final word, namely, “sentence-final negativity (SFN)”. So, is the late AN found in this study similar to the SFN? Hagoort [46] thought that SFN may reflect the sentence-final “wrap-up” processes. Baggio [22] suggested that SFN may reflect the sentence reintegration process. Baggio et al. [38] proposed that the brain supports the recalculation of the discourse model to integrate previous error information. Qiu and Zhou [15] showed that SFN was not affected by the type of disagreeing tense markers in Chinese, but was influenced by the overall reintegration of sentences. Therefore, according to the above researchers’ points of view, we believe that the late negative effect found in this study may also reflect the entire sentence correction process, that is, the sentence-final “wrap-up” processes, which corresponded to the secondary aspect repair process. However, further research is needed to reveal the negative effect.

## 5. Conclusions

This study has investigated the ERP effect of the collocation of verbs with different lexical aspect and aspect markers. The critical words of aspect violation sentences elicited early AN and P600 effects. In the lateral analysis of the post-critical words, we found that the aspect violation sentences led to the late AN. These results revealed the cognitive processing of the collocation of different lexical aspect verb types and aspect markers. The early AN effect elicited by critical words may indicate that when a verb with semantic feature (+punctual) appeared, it triggered the prediction of the aspect marker *le*, but the final one was the aspect marker *zhe*, which led to the wrong prediction, thus producing early AN. At the same time, the clear P600 appeared in the critical word analysis, indicating that the mismatch processing between different lexical aspect verb types and *zhe* was due to syntactic violation. In the analysis of the post-critical words, we found late AN. This negative effect may be attributable to the reinterpretation of aspect and the repair and summary at the end of sentences. Unlike previous studies on Chinese aspect processing, this study’s findings suggest that sentences’ aspect agreement depends not only on the correct combination of aspect markers and temporal words above, but also on the correct collocation of the intrinsic lexical verb types and corresponding aspect markers. ERP itself is related to oscillatory phase [48], and neural oscillation is directly related to language deficit [49]. Therefore, the study of the relationship between neural oscillation phase of aspect violation and language deficit is a promising direction of aspect processing research in the future. We will focus on this research in the future.

## Figures and Tables

**Table 1:** Major ERP components involved in language processing.

ERP Components	Time Course	Distribution	Linguistic Significance
N400	300–600 ms	The central parietal region, the posterior part of the brain and the left and right sides of the brain	Semantic violation
P600	500–900 ms	The posterior part of the brain	Syntactic violation
LAN	300–500 ms	The left anterior part of the brain	Morphosyntactic violation

**Table 2:** Classification of lexical aspect of verbs in Chinese.

	Parameters	(±Dynamic)	(±Punctual)	(±Telic)	Examples	
Types						
State		−	−	−	了解 (know)	喜欢 (love)
Activity		+	−	−	跑 (run)	玩 (play)
Accomplishment		+	−	+	写 (write)	吃 (eat)
Achievement		+	+	+	赢 (win)	到 (arrive)

**Table 3:** Examples of sentences in each condition.

Condition	Example
Correct	安娜/想/着/北京 Anna/xiang/*zhe*/Beijing Anna/miss-*zhe*/Beijing Anna/is missing/Beijing
Aspect violation	安娜/到/着/北京 Anna/dao/*zhe*/Beijing Anna/arrive-*zhe*/Beijing Anna/is arriving/Beijing

## Data Availability

Data available on request due to restriction eg privacy or ethical. The data presented in this study are available on request from the corresponding author. The data are not publicly available due to [Privacy of the Subject].
